# High-Grade Serous Ovarian Carcinoma in the Genomics Era: Current Applications, Challenges and Future Directions

**DOI:** 10.3390/ijms27031617

**Published:** 2026-02-06

**Authors:** Molly Elizabeth Lewis, Chiara Caricato, Hannah Leigh Roberts, Subhasheenee Ganesan, Nadia Amel Seksaf, Eleni Maniati, Michail Sideris

**Affiliations:** 1 Barts and the London School of Medicine and Dentistry, Queen Mary University of London, London E1 2AD, UK; m.e.lewis@smd21.qmul.ac.uk (M.E.L.); n.a.seksaf@smd22.qmul.ac.uk (N.A.S.); 2 Wolfson’s Institute of Population Health, Queen Mary University of London, London EC1M 6BQ, UK; c.caricato@qmul.ac.uk (C.C.); s.ganesan@qmul.ac.uk (S.G.); 3 Barts Health NHS Foundation Trust, London E14 5HJ, UK; hannah.roberts38@nhs.net; 4 Barts Cancer Institute, Queen Mary University of London, London EC1M 6BQ, UK

**Keywords:** high-grade serous ovarian carcinoma, genomics, BRCA, TP53, homologous recombination

## Abstract

High-grade serous ovarian carcinoma (HGSOC) is characterised by profound genomic instability and limited durable responses to standard therapy, leading to poor prognosis. The use of next-generation sequencing technologies has improved understanding of its molecular landscape, revealing consistent Tumour Protein p53 (*TP53*) mutations, homologous recombination defects, pathway alterations, and epigenetic dysregulation. Such genomic profiling now underpins the classification criteria between the ovarian cancer subtypes described by the Cancer Genome Atlas. Widespread chromosomal instability and pathogenic variants in multiple genes distinguish HGSOC from other subtypes of ovarian cancer and, further, from low-grade serous ovarian cancer. Importantly, the new-found understanding of the genomic landscape of HGSOC guides the use of platinum-based chemotherapies and Poly(ADP-ribose) Polymerase (PARP) inhibitors, with homologous recombination deficiency emerging as a cancer vulnerability that enhances treatment response. A combined multi-omics approach integrates transcriptomics, proteomics, metabolomics, and epigenomics to further the understanding of the characteristics, therapeutic targets and treatment resistance within HGSOC. Despite these advances, major challenges persist, including intratumoural heterogeneity and the poor diversity of genomic datasets. Artificial Intelligence (AI) technology, Clustered regularly interspaced short palindromic repeats (CRISPR)-based gene editing, neoantigen-guided immunotherapy and ovarian cancer vaccination indicate a promising future for genomics-guided interventions and support the integration of genomics within multi-omic approaches to improve HGSOC outcomes.

## 1. Introduction

During 2022, ovarian cancer (OC) affected 324,398 women globally, with a mortality of 206,839 [1]. These figures are predicted to rise, reaching over 500,000 cases and over 350,000 deaths globally per annum by 2050 [2].

OC presents with non-specific symptoms [3], and consequently, diagnosis is often made at an advanced stage [4,5,6]. This causes challenges in successful treatment [7] and perpetuates the high mortality rate of 55% at 5 years [4,8]. Traditionally, initial diagnosis is made using imaging in combination with serum CA-125 before definitive diagnosis via surgery and biopsy [5,9]. Treatment approaches involve the use of a combination of radical cytoreductive surgery and platinum chemotherapy [5,6,10].

High-grade serous ovarian carcinoma (HGSOC) accounts for over 70% of OC deaths [6,7]. This subtype of OC is associated with the most germline mutations in homologous recombination DNA repair genes (e.g., *BRCA1/2*) [11] and extensive somatic mutations (e.g., *TP53*) [12].

HGSOC is significantly more genomically unstable than other OC subtypes [5] making it a good candidate for genomics-based risk stratification, diagnostics and therapeutics. This review summarises the most clinically relevant mutations within HGSOC and their applications in clinical practice.

## 2. Genomic Landscape of HGSOC

The characteristic mutation in HGSOC is the *TP53* missense mutation [12]. *TP53* is a tumour suppressor gene (TSG) that acts as a checkpoint to ensure no mutated cells can replicate during cell division [5,6]. Additionally, it is responsible for encoding p53 and is involved in whole-genome duplication [5,6]. As evidenced in Table 1, *TP53* mutations are present in 96% of HGSOC cases [10,12]; the remaining 4% discrepancy could be due to intratumoural heterogeneity and subsequent misclassification as HGSOC [13]. Mutant *TP53* is a highly sensitive marker in HGSOC but relatively non-specific, as it can occur in multiple other cancers [14]. Therefore, clinically, *TP53* mutations have the scope to exclude the diagnosis of HGSOC but not to confirm such a diagnosis. Mutations leading to the gain or loss of function in the *TP53* gene may be the primary driver of chromosomal instability within HGSOC [5,6].

Germline or somatic *BRCA* mutations causing abnormal DNA repair are present in more than 20% of HGSOC cases [5,12,15], as is demonstrated in Table 1. *BRCA1/2* genes are required for the repair of double-stranded DNA breaks through homologous recombination (HRR) [16]. Genetic mutations in *BRCA* prevent the homologous recombination of abnormal DNA strands and lead to the replication of defective DNA within cells; subsequent replication of these cells leads to cancer formation [11,12,15]. Patients with *BRCA1* and *BRCA2* mutations carry a 44% and 17% lifetime risk of HGSOC, respectively [17]. *RAD51* is linked to the Fanconi anaemia BRCA pathway in HGSOC [5]. Similar to *BRCA1/2*, *RAD51* is a tumour suppressor gene responsible for homologous recombination [11,18]. Ergo, both *BRCA* and *RAD51* pathogenic variants contribute to homologous recombination deficiency (HRD) mutational signatures [19], which have been found to be present in as many as 56% of HGSOC patients [20]. Less commonly, deletion mutations within the *PALB2* tumour suppressor gene contribute to *BRCA*-associated HRD mutational signatures [19,21,22,23].

Additional, yet uncommon, pathogenic genomic variations within HGSOC continue to be found, including the *PI3K/Akt/mTOR* [24,25], *FOXM1* [12] and *NOTCH* [12,24] pathway defects, each of which is linked to *BRCA* or *TP53* mutations [5]. Besides this, *CCNE1* is an additional oncogene that plays a role in cell cycle regulation; its amplification encourages tumourigenesis and causes an accumulation of DNA damage [26]. *CCNE1* gene amplification is a marker of primarily early-stage HGSOC and shows an association with *BRCA* [11,12]. *WT1* is a tumour suppressor gene responsible for transcription regulation and cell cycle regulation [27]. Overexpression of the *WT1* gene is associated with *TP53* mutations and leads to a loss of cell cycle regulation and contributes to abnormal proliferation of cells containing DNA damage [28,29].

Genomic alterations can be enhanced and complicated by epigenomic events. DNA methylation of the promoter region is perhaps the most clinically relevant epigenomic aberration [12,30]. Hypermethylation of the promoter region in *BRCA1* can potentially decrease *BRCA1* transcription and is highly associated with HRD in HGSOC [31]. Conversely, widespread *BRCA* hypomethylation increases the oncogene transcription, encouraging HGSOC development [32].

## 3. Current Applications of Genomics in HGSOC

### 3.1. Early Detection and Risk Stratification

Genomics is an effective tool in identifying patient groups at increased lifetime risk of HGSOC (Table 2). Such patients can carry identifiable pathogenic variants in *BRCA1/2*, *RAD51C/D* or other recognised cancer susceptibility genes (CSGs) or are affected by Lynch syndrome and mismatch repair (MMR) genes [21,33,34]. Genetic testing for those genes is routinely offered to those with a personal or strong family history of high-grade epithelial ovarian carcinoma and the Jewish population, as per NICE guidelines [34]. These populations specifically are tested due to a higher incidence of cancer susceptibility genes, particularly BRCA mutations, than the background population. The incidence of BRCA mutations in the Jewish population is 1:40, compared to the UK population risk of 1:250 [35].

Concurrent mutations have a cumulative effect on the relative risk of HGSOC; therefore, polygenic risk scores (PRSs) should be used to estimate the simultaneous effect of single-nucleotide polymorphisms (SNPs) on increasing HGSOC risk [36]. Such calculations are complex, and multiple methods have been proposed without a single validated method being agreed upon [37]. Before integration into routine clinical practice, the polygenic risk score needs to be validated, and its performance optimised for a diverse population, incorporating non-genetic risk factors [36,38].

Liquid biopsy proposes a non-invasive and accessible method for genetic testing of a wider population [5,32]. Liquid biopsies are used to identify and analyse circulating tumour DNA (ctDNA), which is released into the bloodstream after tumour cell apoptosis [39,40]. CtDNA collected via liquid biopsies could be useful, complementary to CA-125, in the diagnosis of HGSOC [40]. Both early and advanced HGSOC involve ctDNA, making it a potential diagnostic biomarker at every stage of the disease [32]. Despite this, little progress has been made to date in this direction towards the early detection of HGSOC. The efforts are still in a primitive stage, with evidence showing significant false positive results [39,40].

Liquid biopsy samples are analysed using next-generation sequencing (NGS)-based approaches. NGS enables highly sensitive detection of tumour-derived alterations in ctDNA, with limits of detection that can reach tumour fractions as low 1 × 10^−5^ [32] and capable of detecting both somatic and germline mutations of a tumour genome [41]. During NGS, multiple genes may be tested at the same time—this can be a sequence of a specific subset of genes or the whole genome [21,41]. There is scope to use NGS in conjunction with the polygenic risk score to evaluate the cumulative risk of cancer susceptibility genes in risk stratification, early detection and potential screening programmes [42]. Facilitating early detection will improve clinical outcomes and mortality [43]. Methylation panels produced from NGS, using ctDNA, also offer accurate diagnostics in both early and recurrent disease, as well as explanations behind treatment response to guide therapies [32].

Cell-free DNA (cfDNA) is an additional promising biomarker in the early detection of HGSOC. cfDNA refers to fragmented DNA circulating in the bloodstream that originates from multiple sources, including normal cells, while ctDNA is the tumour-derived fraction of cfDNA [39,40]. Several studies show the potential utility of cfDNA in identifying *TP53* mutations, which are present in almost all HGSOC cases [10,40,44]. The use of cfDNA is an essential tool for multi-cancer early detection (MCED) trials, in which NGS-based analysis of cfDNA—often incorporating copy number, methylation, or fragmentomic signatures—is used to screen simultaneously for multiple cancer types, within a single assay [45,46,47]. Through the use of positron emission tomography–computed tomography (PET-CT) as well as ctDNA panels [47,48], multi-cancer early detection trials have shown success in the early detection of breast, lung and colorectal cancers [48]. This method of early detection, however, is notably non-sensitive for early HGSOC, limiting its application to HGSOC cases [35].

Risk stratification identifies those at increased risk of HGSOC and facilitates the offer of further care via surveillance every four months [35,49] and individualised risk management for a variety of cancers [47], as well as prophylactic surgery [49]. Serous tubal intraepithelial carcinoma (STIC) represents a well-established precursor lesion for high-grade serous ovarian cancer (HGSOC), supported by genomic, histopathological, experimental and clinical evidence [50,51,52,53,54,55]. Genomic analyses have demonstrated shared TP53 mutations between STIC lesions and concurrent HGSOC, supporting a fallopian tube origin for the majority of HGSOC cases [56]. This paradigm provides the biological rationale for a two-step surgery, early salpingectomy and delayed oophorectomy (RRESDO), as an alternative to conventional risk-reducing salpingo-oophorectomy (RRSO) [49]. Risk-reducing early salpingectomy and delayed oophorectomy is currently only approved for use in research settings until risk reduction is quantified and long-term effects identified [49,57].

### 3.2. Diagnosis and Classification

Understanding the genomic landscape of OC is vital in distinguishing across the several subtypes, based on their serous (HGSOC), clear cell (CCC), mucinous (MC) or endometrioid (EC) nature [58,59]. Diagnosis of the correct subtype is possible based on whether characteristic genomic biomarkers are present or absent. HGSOC can be distinguished from MC and CCC through the detection of *WT1* and from EC through the detection of p53 [58,60]. Furthermore, *TP53* mutations have the scope to differentiate HGSOC from low-grade serous ovarian carcinoma, which itself expresses a high prevalence of *KRAS* and *BRAF* mutations [12,58,61,62].

Molecular diagnostics involve genomics and improve the accuracy of diagnosis and classification beyond that capable with histological and morphological investigations [63,64,65,66,67,68,69,70]. Current techniques of classifying and subtyping HGSOC at the point of diagnosis are limited due to the heterogeneity present within a tumour [71]. Molecular diagnostics using whole-genome sequencing (WGS) could allow for a dualistic classification of HGSOC into ‘star’ and ‘tree’ topologies. A ‘star’ topology demonstrates more heterogeneity, whereas a ‘tree’ topology demonstrates a higher mutation rate [63]. This accounts for two patterns of spatial heterogeneity and allows for a more accurate diagnosis and, therefore, more personalised and effective therapeutics [63]. Molecular testing, however, takes longer than histological diagnosis [72]. Since patient outcomes and mortality are improved with an earlier diagnosis [43], molecular diagnostics may not replace histology as an initial diagnostic tool, but rather complement it to allow personalised approaches.

Using exome capture and sequencing on DNA isolated from 316 HGSOC, TCGA categorised HGSOC into four molecular subtypes: ‘immunoreactive’, ‘differentiated’, ‘proliferative’ and ‘mesenchymal’ [12,64,73,74]. Immunoreactive HGSOC is defined by chemokine (*CXCL10/11* ligand and *CXR3* receptor) expression [12,70] and a high expression of major histocompatibility complex genes and *PDL-1* [65]. Proliferative HGSOC is categorised by increased proliferation marker expression (*MCM2* and *PCNA*) and increased transcription factor expression (*HMGA2* and *SOX11*) alongside reduced ovarian tumour marker expression (*MUC1/16*) [12,70]. Conversely, differentiated HGSOC is categorised by increased ovarian tumour marker expression (*MUC1/16* and *SLP1*) and reduced proliferation marker expression [12,70]. This pattern of expression suggests a more mature stage of development and is generally associated with a worse prognosis [75]. Mesenchymal HGSOC is defined as an increase in stromal components, fibroblasts, and immune response [12,70]. This subtype exhibits a high *CD31* level and *HOX* gene expression compared to other subtypes [12,70]. Survival between subtypes has been found to be statistically different, with immunoreactive providing the best prognosis [75].

### 3.3. Prognostic and Predictive Biomarkers

Genomic profiling has transformed ovarian cancer from a single disease entity into multiple molecularly defined subgroups with distinct prognostic and predictive implications, allowing precision and personalisation. Gene expression signatures, mutational profiles, and copy number patterns capture underlying defects in DNA repair, cell cycle regulation and immune evasion, and these features increasingly guide risk stratification and treatment selection. 

HGSOC is dominated by near-universal *TP53* mutations, whereas *BRCA1/2* alterations, broader HRD, and *PI3K/AKT* pathway changes stratify survival and treatment benefit [76,77,78].

Genomic signatures incorporating *BRCA1/2* status, HRD scores and mutational signatures of defective homologous recombination have been linked with improved progression-free and overall survival, particularly in patients receiving platinum and PARP inhibitors (PARPi). Large-scale sequencing efforts show that approximately 40–50% of HGSOC harbour HRD (germline or somatic *BRCA1/2*, other HRR gene variants, or genomic “scars”), and these tumours display higher initial platinum sensitivity and more durable responses to maintenance of PARP inhibition. Conversely, tumours with intact HRR, frequent *CCNE1* amplification, or *MAPK/PI3K* pathway activation show poorer prognosis and earlier relapse on standard chemotherapy, highlighting the need for alternative targeted approaches [77,78,79].

HRD status represents one of the most clinically advanced predictive biomarkers in high-grade serous ovarian cancer. HRD arises from germline or somatic *BRCA1/2* mutations, alterations in other HRR genes and genomic ‘scars’ that accumulate when double-strand breaks are repaired by error-prone mechanisms. 

ESMO recommendations emphasise that, in current practice, the clinical validity of HRD testing should be judged by its ability to predict benefit from PARP inhibitor (PARPi) maintenance rather than by its fidelity to an ideal biological definition of HRD [80]. Tumours with pathogenic *BRCA1/2* mutations derive the greatest and most consistent benefit from first-line and relapse PARPi therapy, while HRD-positive, *BRCA*-wild-type tumours experience intermediate benefit and HRD-negative tumours demonstrate more modest gains. Current commercial HRD assays based on composite genomic scar scores improve selection for PARPi but imperfectly capture dynamic changes in homologous recombination competence (functional HRD), underscoring the need for functional biomarkers [80,81,82,83].

Beyond HRD, genomic predictors of platinum sensitivity and resistance are particularly critical in a disease where platinum agents remain the backbone of systemic therapy [84]. Tumours exhibiting high genomic instability or loss of homologous recombination proficiency, frequently but not exclusively attributable to *BRCA1/2* dysfunction, demonstrate superior initial response rates and prolonged platinum-free intervals. Conversely, *CCNE1* amplification defines a distinct molecular subset characterised by intact DNA repair, accelerated S-phase entry, and intrinsic platinum resistance. Alterations affecting cell cycle regulators such as *RB1* may further modulate these patterns; notably, *RB1* loss has been implicated in extreme platinum sensitivity within HR-deficient contexts, possibly by amplifying replication stress. Acquired resistance reflects dynamic reconstitution of DNA repair pathways, including secondary “reversion” mutations restoring *BRCA1/2* or other HRR gene function, demethylation of silenced *BRCA1* promoters, and adaptations conferring replication fork stability. Collectively, these events re-establish homologous recombination proficiency, reducing susceptibility to both platinum agents and PARP inhibitors. Comprehensive genomic profiling at relapse, therefore, offers a means to distinguish persistent HRD, which may still respond to DNA-damaging agents, from biologically reconstituted homologous recombination, where alternative strategies are needed [77,78,79,81,85].

### 3.4. Therapeutic Applications

Genomically informed treatment of ovarian cancer increasingly relies on targeting DNA repair defects and oncogenic signalling pathways that are uncovered by NGS to allow precision oncology approaches. It has transformed the therapeutic landscape of HGSOC by enabling biomarker-driven use of PARPi, *PI3K/AKT/mTOR* inhibitors and emerging combinations that exploit DNA repair and signalling vulnerabilities. Parallel advances in immunogenomics and precision oncology frameworks could introduce neoantigen-guided immunotherapy strategies and genomics-driven trial enrolment, especially in recurrent disease [76,78,83,85,86].

As said, PARPi (e.g., olaparib, niraparib, rucaparib) exploit synthetic lethality in tumours with defective homologous recombination, most notably those harbouring pathogenic *BRCA1/2* mutations or high HRD scores. In the first-line setting, PARPi maintenance after platinum-based chemotherapy significantly prolongs progression-free survival in *BRCA*-mutated and HRD-positive patients and is now a core component of standard of care. 

In platinum-sensitive relapse, PARPi maintenance has similarly improved outcomes and allowed a subset of patients to achieve durable treatment-free intervals, although the benefit is attenuated in HRD-negative disease. Combination strategies that pair PARPi with anti-angiogenics or immune checkpoint inhibitors aim to extend efficacy beyond classic HRD-enriched populations by augmenting DNA damage and modulating the tumour microenvironment [78,79,81,82,83].

Beyond PARP inhibition, dysregulation of the *PI3K/AKT/mTOR* pathway offers another rational target, particularly in HGSOC and CCC characterised by *PIK3CA* amplification, *AKT* activation or *PTEN* loss. This pathway drives proliferation, survival, metabolism and angiogenesis, and its activation has been repeatedly linked to poor prognosis and chemotherapy resistance. Preclinical models show that *PI3K*, *AKT* and *mTOR* inhibitors can potentially suppress tumour growth, enhance apoptosis, and restore cisplatin sensitivity, especially when combined with DNA-damaging agents. Early-phase clinical trials of pan-*PI3K* inhibitors, isoform-selective *PI3K* agents, dual *PI3K/mTOR* inhibitors and *mTORC1/2* inhibitors have demonstrated modest single-agent activity but significant toxicity, prompting development of biomarker-enriched designs that select patients based on *PIK3CA* mutations, *PTEN* loss or pathway activation signatures. There is growing interest in combining *PI3K/AKT/mTOR* inhibitors with PARPi, exploiting crosstalk between DNA repair and survival signalling to overcome resistance and widen the pool of patients who derive meaningful benefit from targeted therapy [78,82,87,88,89].

Immunogenomics introduces an additional therapeutic dimension by linking tumour mutational and neoantigen landscapes to response to immune checkpoint blockade. OC with HRD or ultra-mutated profiles generate higher neoantigen loads, which may increase T-cell recognition and make these tumours more susceptible to *PD-1/PD-L1* or *CTLA-4* inhibitors when appropriately primed. In parallel, genomic instability can upregulate interferon and STING pathway signalling, further promoting an inflamed tumour microenvironment that favours immune checkpoint activity [86,90,91,92,93].

Whole-exome and RNA sequencing can be used to predict patient-specific neoantigens, quantify tumour mutational burden and characterise immune gene expression signatures, enabling identification of “hot” tumours with T-cell-inflamed microenvironments as candidates for checkpoint inhibition or adoptive cell therapy [92]. Conversely, tumours with low mutational burden and “cold” immune profiles may require combination approaches that enhance immunogenic cell death, such as pairing PARPi or *PI3K/AKT/mTOR* inhibitors with *PD-1/PD-L1* antibodies, anti-angiogenic agents or radiation. Preclinical data indicate that PARP inhibition can increase cytosolic DNA, activate cGAS–STING signalling and upregulate *PD-L1*, providing a rationale for PARP–checkpoint combinations [93]. On the other hand, *PI3K* pathway blockade may deplete immunosuppressive myeloid and regulatory T-cell populations and potentiate vaccine or checkpoint responses [82,93]. Ongoing basket and umbrella trials incorporating genomics and immunogenomic profiling aim to refine patient selection for these combinations and to define robust biomarkers of durable immune response in ovarian cancer [87,93].

At present, immunotherapy in ovarian cancer is best considered in the context of early clinical trials, especially for combinations built on genomic and immunogenomic profiling. Outside of trials, checkpoint blockade is mainly reserved for rare MSI-H/dMMR cases or when used according to tumour-agnostic approvals, while vaccines and adoptive cell therapies remain experimental [94].

Within this framework, antibody–drug conjugates such as mirvetuximab soravtansine (MIRV) provide an additional immunogenomically relevant tool by exploiting surface antigen expression defined by tumour genomics and epigenetics. The MIRASOL [95] and PICCOLO [96] trials in high-FRα platinum-resistant and platinum-sensitive ovarian cancer demonstrated that rigorous biomarker selection using standardised FRα immunohistochemistry enriches results for patients, who get substantial benefit from mirvetuximab, with improved survival and patient-reported outcomes compared with chemotherapy in the resistant setting. These studies illustrate how integrating surface antigen profiling into molecular work-ups can stratify patients for antibody–drug conjugate-based strategies and offer a platform for future combinations in which mirvetuximab-induced immunogenic cell death is paired with checkpoint inhibitors, guided by concurrent assessment of FRα expression, HRD status and immune-inflamed gene signatures [95,96,97,98,99,100].

Longitudinal genomic profiling, including secondary *BRCA* or other HRR gene reversions, PARP1 mutations, replication fork stabilisation, upregulation of alternative DNA repair pathways, and activation of survival signalling such as *PI3K/AKT* and *RAS/MEK/ERK*, can uncover drug resistance mechanisms. Serial and multi-region tumour sampling as well as liquid biopsies can track these evolutionary changes, enabling adaptive therapy—such as switching from PARPi to *ATR/WEE1* inhibition in HRR-restored disease or adding *PI3K/MEK* blockade in pathway-addicted clones. Such adaptive designs are increasingly built into clinical trials aimed at intercepting resistance at molecular progression rather than radiologic relapse [79,82,87,101,102].

Precision oncology initiatives using targeted NGS panels or WGS in HGSOC have shown that a high proportion of patients harbour at least one actionable alteration, most commonly in *BRCA1/2*, other HRR genes, *PI3K/AKT/mTOR* components, or rare fusions and receptor tyrosine kinase mutations. Prospective molecular tumour boards can translate these findings into matched therapies within umbrella or basket trials, improving access to PARPi, *PI3K/AKT/mTOR* inhibitors, *NTRK/RET/ALK* inhibitors, or immune checkpoint blockade in biomarker-selected subsets. The integration of genomic profiling into future routine care thus underpins an ongoing shift from empiric chemotherapy toward a personalised, stratified treatment paradigm in ovarian cancer, with current efforts focused on harmonising testing, improving trial accrual, and generating real-world evidence on long-term outcomes [77,85,101].

## 4. Integration of Multi-Omics and System Genomics

While genomics offers substantial insight for diagnosis, treatment, and prevention of OC, genomic information alone cannot fully explain tumour behaviour, heterogeneity, or disease evolution. Multi-omics approaches integrate genomic data with other high-dimensional molecular layers at bulk, single-cell or spatially resolved levels, including transcriptomics, proteomics, metabolomics, and epigenomics, to provide a more comprehensive view of cellular and tissue functions [11,12,103,104]. By capturing regulatory and functional changes beyond DNA sequence variation, multi-omics analyses improve our understanding of how genetic alterations influence gene expression, protein activity, metabolic pathways, and epigenetic regulation. This integrative framework is particularly valuable for elucidating the HGSOC evolution and for linking genomic mutations to downstream functional consequences that drive tumour progression and therapeutic response.

Large-scale proteomic and proteogenomic analyses, including those generated through the Clinical Proteomic Tumour Analysis Consortium (CPTAC), provided comprehensive proteogenomic characterizations of HGSOC, demonstrating that widespread copy number alterations and ubiquitous TP53 mutations lead to highly heterogeneous protein expression and signalling outcomes [103,105,106]. CPTAC identified proteomic subtypes of HGSOC associated with differences in immune signalling, metabolic activity, and patient prognosis, refining classifications previously defined by transcriptomics. Importantly, these analyses showed that only a subset of genomic alterations are consistently propagated to the protein level, highlighting extensive post-transcriptional and post-translational regulation in HGSOC [103]. More recently, Chowdhury et al., in a mass spectrometry-based proteogenomic analysis of 242 HGSOC tumours, identified and validated a 64-protein signature that predicts with high specificity a subset of HGSOC tumours refractory to initial platinum-based therapy [106]. A subtype of proteomics, phosphoproteomics, describes post-translational phosphorylation of proteins and has helped identify key phosphorylated sites underlying ovarian cancer, providing further potential therapeutic targets and biomarkers [107]. Four glycolytic enzymes, PFKFB2, PFKL, ALDOA, and TPI1, related to fructose and mannose metabolism have been found to promote ovarian tumour growth [107]. Differentially expressed kinases, also phosphoproteins, whose dysregulation is related to cancer growth, are also targets in many therapies. Another post-translational modification in ovarian cancer identified through proteomics is ubiquitination. One example where this occurs is in the extracellular signal-regulated kinase (ERK) pathway, where aberrant activation increases resistance to chemotherapy and tumourigenicity as described by Rao et al., providing another potential therapeutic target [108].

Metabolomics refers to the study of genomics on metabolites and other substances involved in cellular metabolism. The study of these metabolites and their effects is important in OC, as it has known mutations that impact its metabolic phenotypes. Squalene epoxidase (SQLE), an enzyme involved in cholesterol metabolism, has been found to be highly expressed in HGSOC, driving cancer cell proliferation by inhibiting apoptosis, and is related to both peritoneal metastasis and poor prognosis in these patients. This represents a potential clinical biomarker for predicting prognosis in HGSOC [109]. HRD tumours have increased expression of the oxidative phosphorylation (OXPHOS) pathway and, therefore, are more sensitive to inhibitors of OXPHOS, including metformin [110]. Understanding the metabolomics of these tumours suggests a new mechanism to help determine, in a more personalised way, tumour sensitivity to PARPi treatment and potentially introduce existing drugs, like metformin, which could synergically increase PARPi-induced apoptosis.

Whilst metabolomics is an exciting area of potential biomarker development, large-scale and well-validated metabolomics studies in ovarian cancer remain limited, and no consensus has been reached yet on a group of metabolite biomarkers for use in detecting OC [111]. As described [112] by Warburg et al., cancer cells primarily use anaerobic glycolysis to produce energy, even in the presence of oxygen. This mechanism has been described in OC, resulting in increased glucose 6-phosphate and glucose 1-phosphate in malignant ascites and cancer tissue [111], suggesting a potential target for a biomarker to aid diagnosis. Chemoresistance in cancer cells has also been linked with increased fatty acid oxidation, causing increased expression of uncoupling proteins. This subsequently results in higher levels of carnitine proteins, which has been found in the serum and tissue of OC patients [113]. Another metabolite, N1,N12-diacetylspermine, part of the polyamine metabolite pathway, has also been found in higher concentrations in tissue, plasma, urine and saliva in patients with OC [114].

The use of multi-omics can help integrate this knowledge; some examples of this include *BRCA1* promoter methylation, which affects the mRNA expression of the gene and impairs its detection in usual assay testing. This is thought to occur in 10–15% of HGSOC, where an effort to refine assays to better identify patients with OC related to silenced *BRCA1* genes will translate into direct patient benefit [115]. Adjacent to this, the KOMET study has found that the *BRCA1* promoter methylation is associated with better response to PARPi and platinum chemotherapy than tumours without methylation or classical HRD; therefore, detection of methylation could be considered as part of the work-up prior to initiation of a PARPi [116].

Transcriptomics has also identified highly expressed genes in ovarian cancers, such as cyclins like *CCNE1,* which in turn lead to elevated cyclin E protein and phospho-CDK2 pathway activity. This signature is seen in the proliferative subtype of HGSOC, previously defined by TCGA. Highly expressed *CCNE1* has been associated with a poor prognosis and platinum resistance, providing useful prognostic information but also providing further therapeutic targets [117]. It has also been suggested that tumours found to have *CCNE1* amplification may benefit from CDK2 inhibitors [118]. Moreover, integration of WGS with single-cell transcriptomics, digital histopathology and multiplexed immunofluorescence implicates anatomical sites and mutational processes as key determinants of intratumoural phenotypic divergence and immune resistance mechanisms in HGSOC [104].

Chemotherapy with platinum-based agents, e.g., cisplatin, carboplatin, is a major part of treatment for many OC cases; however, over time tumours can develop resistance. Multi-omics has helped identify changes in gene expression relating to cellular metabolism, and how these changes can contribute to platinum resistance, including through the loss of hypermethylation, OXPHOS and fatty acid oxidation [119,120]. These are potential therapeutic targets to prevent emerging resistance to platinum-based chemotherapy [121].

Multi-omics integration has a clear and important role in advancing our understanding of OC development, and as demonstrated above, it can reveal new therapeutic targets, potential biomarkers, and treatment resistance mechanisms and explain tumour progression and metastasis. Integration of these large, complex molecular datasets is technically challenging due to the high dimensionality, heterogeneity, and frequently missing values across the data types. Computational methods developed to integrate multi-omics utilise statistical and machine learning approaches ranging from canonical correlation analysis and matrix factorization to machine learning, artificial neural networks and, more recently, deep generative learning [122]. Classic network-based approaches are ideal for analysing gene regulatory and protein–protein interaction (PPI) networks, and have been used to identify biomarkers in ovarian cancer. For example, weighted gene co-expression network analysis (WGCNA) has been used to map out transcriptional programmes in HGSOC [123] and has helped identify platinum resistance modules [124]. PPI networks show potential for identifying biomarkers [125] and can elucidate how genetic mutations rewire intracellular signalling networks [126].

Machine learning, in particular, the recent development of deep learning methods, can uncover additional prognostic and biological information embedded within and across these heterogeneous multi-dimensional data types [127]. This further translates to better prediction of treatment response, the modelling of chemotherapy resistance or the creation of gene expression sensitivity profiles to predict tumour responsiveness to PARPi [128]. Importantly, molecular data can now be further integrated with additional modalities such as radiological and histopathological images, as well as clinical features, improving prognostic accuracy compared to conventional statistical models [129]. There are many examples of computational tools that have been created for this purpose, one of which is subtype-GAN—a deep learning tool created to integrate genomic data [130]. Finally, beyond purely data-driven approaches, artificial intelligence (AI) frameworks can incorporate systems biology models and curate biological knowledge from public databases, enabling the integration of prior biological structure to interpret molecular interactions and system-level behaviour [131,132].

## 5. Challenges and Limitations of Genomics in HGSOC

Despite its great potential in aiding the clinical understanding and care behind HGSOC, there are limitations within genomics that restrict clinical applications and act as barriers in genomic integration into standards of care. First and foremost, cost implications can limit genomic applications to healthcare systems that can sustain time and resource allocations. With rapidly evolving technology and cost drop, this will hopefully be less of a challenge in the coming years.

Accurate classification and effective targeted therapeutic use are limited by intratumoural heterogeneity. This heterogeneity refers to variation in cell genotype and phenotype within a single tumour and is a hallmark of cancer [133,134]. Heterogeneity within a tumour can be temporal as well as spatial, meaning that mutation variety may be present from the commencement of the tumour development or could develop during tumour progression, respectively [135]. This introduces ambiguity to tumour classification [63] affecting the success of subtype-specific therapies [133]. TGCA proposed that patients with a lack of *TP53* mutations tested this way due to intratumoural heterogeneity and their diagnosis was, therefore, not purely HGSOC [12,13]. Each cell in a tumour may react differently to treatment: one cell may respond to platinum chemotherapy, whilst another may be platinum resistant [134,135]. Drug failure across some cells of the tumour but not others means that multiple therapies with different mechanisms of action in different genomic targets are required to treat a single tumour [133]. This leads to a poor prognosis as it is more difficult to create an effective treatment [133,134]. This is a particularly significant issue within platinum and PARPi resistance [106,135]. Tracking and understanding heterogeneity can be completed using AI to analyse imaging, an integrated multi-omics approach or cfDNA analysis [134] which was discussed in previous sections. Methods of refining tumour classification have been proposed using a dualistic approach, accounting for the heterogeneity within the tumour [63]. However, the only definitive solution to intratumoural heterogeneity is invasive surgery rather than current genomic therapies [135].

Although helpful in identifying biomarkers in HGSOC, sequencing the genome is an expensive process. In the initial stages of development, whole genome sequencing was reducing in price; however, that has now plateaued [136]. This reduces the accessibility of lifesaving diagnostic tools for those in socio-economic deprivation. Insurance contributions help to bridge the gap and improve access; however, this is inadequate [136]. The Detect-2 trial has taken another approach to improving access: this clinical trial offers simple at-home genetic testing to those already diagnosed with HGSOC using saliva, which is as effective as blood testing [137,138], in the hope of increasing patient participation with genetic testing [139].

Other inequalities are significant in the application of genomics in HGSOC. Approximately 80% of whole-genome sequencing has involved European patients of white or Caucasian ethnicity [136]. This is not representative of the diverse global population; therefore, the findings and applications within genomics may not be applicable to non-white individuals [136]. Without diversifying the pool of participants in genome testing, the genomic landscape of HGSOC cannot be universally understood, and therefore, genomics cannot be integrated into the standard of care [136,140]. Racial bias in results limits the use of the polygenic risk score as a screening tool [36].

Identifying high-risk genetic mutations, especially those that are hereditary, carries ethical implications. Notification of carrying a high-risk mutation is not the same as receiving a confirmed diagnosis; there is a chance the patient will not be affected. Alerting someone to their risk of HGSOC may relieve anxiety, as they can undergo risk-reducing salpingoophorectomy to reduce the risk. Others find the risk of cancer, and the idea of risk-reducing salpingoophorectomy, anxiety-inducing, especially prior to starting their family [141,142]. High-risk patients also harbour this anxiety for their family members [142]. With hereditary mutations, confidentiality is put at risk. First-degree relatives of a high-risk patient could be affected; however, disclosure of such information would be a breach of confidentiality. The alternative to this is withholding information that could empower family members to seek genetic testing and risk-reducing options [143].

Discoveries of novel genomic targets require pure samples [144] processed via standardised and validated methods [145,146,147]. Standardisation of sample collection and analysis requires strict protocols followed by each researcher [49,146]. This includes using the SEEFIM (Sectioning and Extensively Examining the FIMbria) protocol for dissecting patient samples for analysis of STIC [49]. Following such protocols proves to be challenging across multiple sites, and many studies struggle with protocol deviations, leading to inconsistency in methods of sample collection and analysis [146,148,149].

WGS and NGS produce a huge amount of genomic data, which must be analysed and interpreted [136]. This process requires intensive and difficult training, leading to issues such as inaccurate diagnosis of genomic mutations through human error and lack of proper training [136]. Recently, the emergence of population genome databases has made this information more readily available, so less interpretation is required [136,150]. This makes whole-genome sequencing and interpretation much easier, more accessible and more accurate; however, it introduces the possibility of overinterpretation [150].

## 6. Future Perspectives

AI and machine learning is an exciting area of development within genomics in OC. Improvements in the interpretation of NGS and WGS using AI will allow for better use and understanding of multi-omics. The continuous advancement of integrative and AI-based approaches requires coordinated efforts to generate large, diverse, clinically well-annotated datasets that support reproducibility, generalizability, and clinical translation. Combining imaging and multi-omics will be an important future research topic with limited current literature, improving outcomes by providing a better understanding of the heterogeneity of OC [151]. AI may also be applied in the diagnosis and screening of ovarian cancer, with an example being the use of cfDNA high-risk methylation biomarkers [152]. Deep learning has also been trialled in predicting treatment response by detecting HRD mutations, which alter response to platinum therapies [153].

Other future endeavours include gene editing. Patients with known high-risk pathogenic variants could have their risk profile altered using this technology. CRISPR (Clustered regularly interspaced short palindromic repeats) technology could be used to carry out targeted deletions within these genes using Cas9 [151]. *CPT1A* is an enzyme involved in the rate-limiting step of fatty acid oxidation, desensitising high-grade ovarian cancer cells to platinum-based chemotherapy agents [152]. It was identified by multi-omics as a potential therapeutic target to increase sensitivity to platinum therapies [152], and the utilisation of CRISPR to knock out *CPT1A* could be a potential future therapy in these cases [152]. CRISPR knockout of *BRCA1* in ID8 cells also increases platinum and PARPi response [152].CRISPR technology has also identified *PCTM1*, which is overexpressed in late-stage metastatic tumours and appears to increase distant metastasis and ascites formation, as a potential therapeutic target in OC [152].

A group of researchers based in Oxford have received funding to begin development on the first OC vaccine [154]. This is being developed using mRNA-based technology and initially aimed at those with recognised high-risk pathogenic variants with the aim of preventing HGSOC from occurring.

There are also prospects for the introduction of new population-level genomic screening, the feasibility of which is currently being evaluated in trials such as Detect-2 [155]. This plans to investigate whether direct-to-patient genetic testing is feasible in terms of patient satisfaction, psychological outcomes, and economic analysis. This information can directly impact future clinical care, as well as identify those who are at high risk. A known *TP53*, *BRCA* or *RAD51* pathogenic variant indicates the patient is at high risk of developing HGSOC originating in the fallopian tube [10,35,57].

## 7. Conclusions

**Figure 1:**
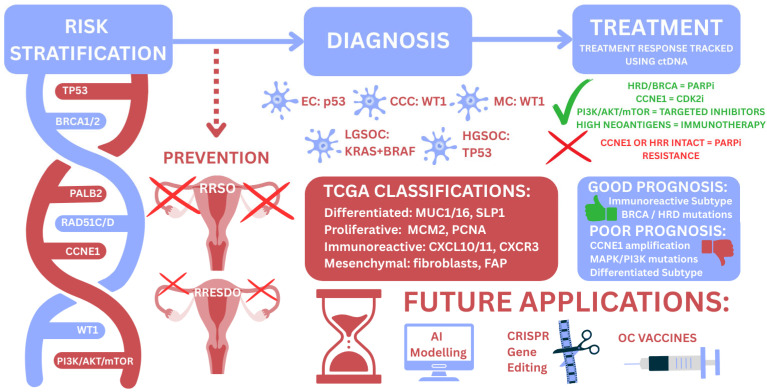
An infographic summary of the current applications of genomics in HGSOC. RRSO = Risk-reducing salpingo-oophorectomy; RRESDO = Risk-reducing early salpingectomy and delayed oophorectomy; EC = Endometrioid carcinoma; CCC = Clear cell carcinoma; MC = Mucinous carcinoma; LGSOC = Low-grade serous ovarian carcinoma; HGSOC = High-grade serous ovarian carcinoma; TCGA = The Cancer Genome Atlas; ctDNA = Circulating tumour DNA; HRD = Homologous recombination deficiency; HRR = Homologous recombination repair; AI = Artificial intelligence; CRISPR = Clustered regularly interspaced short palindromic repeats; OC = Ovarian cancer.

Much of the current scope and future applications of genomics within HGSOC is summarised in Figure 1. Genomic technologies are already reshaping the management of HGSOC, particularly in risk assessment, prognostication, and the selection of targeted therapies such as PARPi. In the coming years, genomics has the potential to extend its impact into early detection, definitive diagnosis, and the development of curative strategies. Emerging tools—including CRISPR-Cas-based editing—offer the prospect of truly personalised precision therapies tailored to each tumour’s molecular profile.

To realise this potential, future research must prioritise the integration of genomics with complementary multi-omics approaches, enabling less invasive and more accurate alternatives to traditional diagnostic and therapeutic methods. Equally important is addressing critical challenges such as intratumoural heterogeneity and the limited representation of diverse populations in current genomic datasets. Overcoming these barriers will be essential in ensuring that genomics-driven advances translate into effective, equitable care for all patients with HGSOC.

## Figures and Tables

**Table 1:** Key genomic alterations in HGSOC and their clinical relevance.

Gene(s) or PathWays	Type of Alteration(s)	Frequency in HGSOC	Normal Biological Role	Clinical Implications
*TP53*	Missense, Loss of Function	>96%	Cell Cycle Checkpoint	Diagnosis (Non-Specific)
*BRCA1/2*	Germline or Somatic Loss of Function	>20%	HRR	HRD Signature, Treatment (PARPi) Response
*RAD51C/D*	Germline or Somatic	3–5%	HRR	HRD Signature, Treatment (PARPi) Response
*PALB2*	Germline, Deletion	<1%	BRCA Tethering	HRD Signature
*CCNE1*	Amplification	15–20%	Cell Cycle Progression	Treatment (Platinum) Response
PI3K/AKT/mTOR	Activation	~45%	Cell Growth	Targeted Therapy (e.g., Temsirolimus, Everolimus)
*WT1*	Overexpression	Up to 97%	Mullerian Lineage Marker, Cell Cycle Regulation	Diagnosis
*BRCA1, RAD51C*	Promoter Hypermethylation	10–15%	HRR	HRD Signature, Treatment (PARPi) Response

HRR = Homologous recombination repair; HRD = Homologous recombination deficiency; PARPi = Poly ADP-Ribose Polymerase inhibitor.

**Table 2:** Clinical applications of genomics in HGSOC.

Application	Marker(s)	Strengths	Limitations
Risk Stratification	*BRCA1/2* and *RAD51C/D* testing, PRS	Identify high-risk individuals, increase access to RRSO/RRESDO	Limited diversity in PRS data, PRS not yet validated
Early detection	ctDNA methylation panels	Minimally invasive, potential adjunct to CA-125	Low sensitivity in early disease, no screening programme, protocol deviations
Diagnosis and Classification	*TP53, WT1, KRAS, BRAF, CXCL10/11, CXCR3, PDL-1, MCM2, PCNA, HMGA2, SOX11, MUC1/16, SLP1, HOX*, fibroblasts	Improvement upon histotype classification accuracy, molecular subtyping	Time-consuming, expensive, requires sequencing, intratumoural heterogeneity
Prognostic Markers	HRD score, *CCNE1* amplification, copy number instability	Predicts platinum and PARPi response and outcome	Variability in HRD testing
Therapeutic Guidance	*BRCA1/2*, HRD, neoantigen load, *PI3K/AKT/mTOR*	Enables precision treatment, more personalised medicine	Resistance evolution, expensive, intratumoural heterogeneity
Monitoring and Resistance	ctDNA tracking, reverse mutation sequencing	Detects molecular progression	Not yet standardised

PRS = Polygenic risk score; RRSO = risk-reducing salpingo-oophorectomy; RRESDO = risk-reducing early salpingectomy and delayed oophorectomy; ctDNA = circulating tumour DNA; PARPi = Poly ADP-Ribose Polymerase inhibitor; HRD = Homologous recombination deficiency.

## Data Availability

Data sharing is not applicable. No new data were created or analysed in this study.

## Abbreviations

The following abbreviations are used in this manuscript:

AI	Artificial Intelligence
CCC	Clear Cell Carcinoma
cfDNA	Cell-Free DNA
CPTAC	Clinical Proteomic Tumour Analysis Consortium
CRISPR	Clustered Regularly Interspaced Short Palindromic Repeats
CSG	Cancer Susceptibility Genes
ctDNA	Circulating Tumour DNA
EC	Endometrioid Carcinoma
HGSOC	High-Grade Serous Ovarian Carcinoma
HRD	Homologous Recombination Deficiency
HRR	Homologous Recombination Repair
MC	Mucinous Carcinoma
MCED	Multi-Cancer Early Detection
MMR	Mismatch Repair
NICE	National Institute for Health and Care Excellence
NGS	Next-Generation Sequencing
OC	Ovarian Cancer
OXPHOS	Oxidative Phosphorylation
PARPi	Poly ADP-Ribose Polymerase inhibitor
PPI	Protein–Protein Interaction
PRS	Polygenic Risk Score
RRESDO	Risk-Reducing Early Salpingectomy and Delayed Oophorectomy
RRSO	Risk-Reducing Salpingo-Oophorectomy
SEEFIM	Sectioning and Extensively Examining the Fimbria
SNP	Single-Nucleotide Polymorphism
SQLE	Squalene Epoxidase
STIC	Serous Tubal Intraepithelial Carcinoma
TCGA	The Cancer Genome Project
